# Molecular Composition, Seasonal Variation, and Size Distribution of n-Alkanes, PAHs, and Saccharides in a Medium-Sized City of Guanzhong Plain, Northwest China: Evaluation of Control Measures Executed in the Past Decade

**DOI:** 10.3390/toxics11020164

**Published:** 2023-02-09

**Authors:** Bianhong Zhou, Qiao Feng, Chunyan Li, Lihua Jiao, Kaijing Cheng, Steven Sai Hang Ho, Zhongtao Wen, Jianjun Li

**Affiliations:** 1 Shaanxi Key Laboratory of Disaster Monitoring and Mechanism Simulation, College of Geography and Environment, Baoji University of Arts and Sciences, Baoji 721013, China; 2 State Key Laboratory of Loess and Quaternary Geology, Key Lab of Aerosol Chemistry and Physics, Institute of Earth Environment, Chinese Academy of Sciences, Xi’an 710061, China; 3 Division of Atmospheric Sciences, Desert Research Institute, Reno, NV 89512, USA; 4 Baoji Ecological Environment Science and Technology Service Center, Baoji 721000, China

**Keywords:** n-alkanes, saccharides, PAHs, organic tracers, particle size distribution, pollution reduction

## Abstract

Baoji is a medium-sized city in the Guanzhong Plain of northwest China. The compositions of three important organic groups, namely n-alkanes, polycyclic aromatic hydrocarbons (PAHs), and saccharides in atmospheric aerosol with different aerodynamic diameters in power were determined. Both seasonal and daily trends of the target organic chemical groups were demonstrated. The concentration levels of total quantified n-alkanes and saccharides in total suspended particles (TSP) in winter were 541 ± 39 and 651 ± 74 ng·m^−3^, respectively, much higher than those of the other three seasons. A high total quantified PAHs concentration level of 59.6 ± 6.4 ng·m^−3^ was also seen in wintertime. n-Alkanes showed a bimodal percent distribution in spring, autumn, and winter. Two peaks were found with the particle sizes of 0.7 μm < *Dp* < 2.1 μm and 3.3 μm < *Dp* < 4.7 μm, respectively. In summer, a unimodal was seen with a peak of 4.7 μm < *Dp* < 5.8 μm. Dehydrated saccharides and PAHs present a unimodal size distribution peaking at the aerodynamic diameters of 0.7 µm < *Dp* < 2.1 µm. In contrast to glucose and fructose, they mainly exist in the coarse mode particles and have the highest concentrations at aerodynamic diameters of 4.7 µm < *Dp* < 9.0 µm. The geometric mean diameters (GMD) of n-alkanes and saccharides of the fine particles in winter were higher than in the other seasons. Compared with the data in 2008, the fossil fuel-derived n-alkanes and PAHs in winter decreased by nearly an order of magnitude in 2017. Both the carbon preference index (CPI) of n-alkanes and the diagnostic ratios of PAHs suggest that coal combustion and vehicle exhaust were the major pollution sources of the organic groups in the two decades. It should be noted that the contribution of traffic emissions greatly increased from 2008 to 2017, consistently with a large raise of registered vehicles in Baoji city. The overall results confirm that the control measures conducted by the local government in the recent decade mitigated the air pollution in this city.

## 1. Introduction

Atmospheric organic aerosols pose important impacts on both regional and global climate, hydrological cycle, and human health [1,2]. Their influence generally depends on their particle size, concentration level, and chemical composition [3,4]. The scattering and light absorption of atmospheric particles greatly vary with their particle sizes. In particular, the fine particles have greater scattering, absorption efficiency, and stronger climate impact [5]. Furthermore, they could reach deep into the lungs, while the ultrafine size particles could even enter the blood circulation and cause cardiovascular diseases, leading to important harm to human health [6].

Fossil fuel combustion, mostly including coal burning and vehicular combustion, is an important source of organic aerosols in urban Chinese cities [7]. For organic aerosols, n-alkanes, polycyclic aromatic hydrocarbons (PAHs), and saccharides are three major components. n-Alkanes and PAHs are known as important non-polar compounds. Fossil fuel combustion and biomass combustion are the main sources of anthropogenic n-alkanes, while plant wax emissions, pollen, and microorganisms release natural n-alkanes [8]. PAHs are carcinogenic, teratogenic, mutagenic, and genotoxic, which are seriously harmful to human health [9,10]. They mainly are associated with fine particulate matter (PM) in the atmosphere [11]. Particularly, higher molecular weight (HMW) PAHs (i.e., a higher number of aromatic rings) are more toxic than the lower (LMW) ones [12,13]. Dehydrated saccharide was the most abundant organic component in rural areas of Guanzhong Plain, China, where the atmospheric aerosols are influenced by residential biomass burning activities [14]. Saccharides originated from fungal spores, pollen, plant fragments, bacteria, and viruses which have been proven to be important water-soluble organic compounds and biological tracers in environmental aerosols [5,15]. Therefore, the study of concentration levels, chemical composition, and particle size distribution is of great significance for identifying the source and establishing pollution control measures. 

Baoji was located at the westernmost end of the Guanzhong Plain, which was a transmission terminal of heavy pollution in this region. Surrounded by mountains in the south, west, and north, (especially blocked by the tall Qinling Mountain in the south), typical valley topography and perennial high relative humidity (RH) lead to difficulty in pollutant dispersion. In recent decades, the central and local governments in China have implemented a series of actions to reduce air pollution in the country. Especially since 2013, when the Chinese State Council quickly released the ‘Atmospheric Pollution Prevention and Control Action Plan’, intensive emission control strategies have been conducted that led to the significant improvement of air quality in China [16]. The mass concentration of PM_2.5_, PM_10,_ and SO_2_ showed an obvious decrease after 2013 in Baoji (Figure S1). Compared with 2008, the mass concentration of PM_10_ and SO_2_ in 2017 decreased by 76.4% and 47.8%, respectively [17]. Wang et al. and Xie et al. determined the characteristics of n-alkanes and PAHs in Baoji city in the spring and winter of 2008 [18,19]. However, due to changes in energy production structure and emission sources, the air pollution characteristics must be changed accordingly, thus further investigation was required. In this study, a multistage impactor sampler was used to collect atmospheric PM samples in Baoji city. The concentration levels and particle size distributions of n-alkanes, PAHs, and saccharides were determined. The pollution sources of the organic aerosols were apportioned and discussed. Lastly, by comparing with the data obtained in 2008, we have assessed the efficiency of the pollution control policies implemented by the local government throughout the past ten years. 

## 2. Experimental Section

### 2.1. Sample Collection

The sampling site was located on the roof of the Mingli Building of the Baoji College of Arts and Sciences. The area was locally surrounded by commercial and residential buildings and a cultural parish. The sampling port was set at 1.5 m over the rooftop which was covered with a waterproof linoleum to prevent the influence of secondary dust. No obvious pollution source was found around. Before sampling, all collection filters (quartz fiber, o.d. = 80 mm) of 80 mm (Whatman, QM/A, Maidstone, England, UK) were pre-heated in a furnace at 450 °C for 6 h. Before and after the sampling, the filters, equilibrated in a chamber at a temperature of 22 ± 2 °C and RH of 30–40%, were weighed with a microbalance (BT125D, Sartorius, Gottingen, Niedersachsen, Germany). The PM samples were collected using a multistage impingement atmospheric particle sampler (Tisch Environmental Inc., Cleves, Ohio, USA) running at the flow rate of 28.3 L/min. Each sample set comprised nine filters and included Stage A: (*Dp* < 0.43 μm), Stage B: (0.43 μm < *Dp* < 0.65 μm), Stage C: (0.65 μm < *Dp* < 1.1 μm), Stage D: (1.1 μm < *Dp* < 2.1 μm), Stage E: (2.1 μm < *Dp* < 3.3 μm), Stage F: (3.3 μm < *Dp* < 4.7 μm), Stage G: (4.7 μm< *Dp* < 5.8 μm), Stage H: (5.8 μm< *Dp* < 9.0 μm), and Stage I: (*Dp* > 9.0 μm). Samples were collected in spring (18 April to 8 May 2017), summer (15 July to 4 August 2017), autumn (15 October to 5 November 2017), and winter (15 December 2017 to 2 January 2018). At the same time, the daily samples were collected based on daytime (08:30–20:00) and nighttime (20:00 to 08:30 the next day). Three sets of membranes were collected day and night in each season, and each set was collected for seven days. All PM-loaded filter samples were packed in pre-baked aluminum foil and stored in a freezer at −24 °C until the chemical analyses. Meteorological parameters were collected during the sampling event and are listed in Table S1.

### 2.2. Sample Extraction, Derivatization, and Chemical Analysis

#### 2.2.1. Extraction and Derivatization

A quarter of the filter was spiked with internal standard (IS) and extracted with a mixture of dichloromethane and methanol (2:1, *v*/*v*) under ultrasonication three times (each time for 15 min). After extraction, the extracts were concentrated to 100 μL using a rotary evaporator under vacuum and then dried under a high-purity nitrogen stream. Then, 50 μL of *N*,*O*-bis-(trimethylsilyl) trifluoroacetamide (BSTFA) with 1% trimethylsilyl chloride and 10 µL of pyridine) was added into the concentrated extract in a capped vial and heated in a block at 70 °C for 3 h. When the solution cooled down, 40 μL of tridecane was added as injection IS. More detailed sample pretreatment steps are shown in [20].

#### 2.2.2. GC/MS Analysis

After the sample pretreatment, the n-alkanes, saccharides, and PAHs in the extract were analyzed by a gas chromatography/mass spectrometer (GC/MS, 7890A GC/5975C MSD, Agilent Technology, Santa Clara, CA, USA). The analytes were separated with a DB-5MS fused silica capillary column (Agilent Technology, 25 m × 0.25 mm i.d., 0.5 µm film thickness) with the GC oven temperature programmed at an initial temperate of 50 °C for 2 min, ramped to 120 °C at 15 °C min^−1^ and then increased to 300 °C at 5 °C·min^−1^, and held at the final temperature for 16 min. One microliter of the extract was injected in a spitless mode at an injector temperature of 280 °C and scanned from 50 to 650 Daltons using electron impact (EI) mode at 70 eV. A total of 19 n-alkanes, 14 PAHs, and 10 saccharides were quantified in this study. The average extraction recoveries of the target compounds were between 70% and 120%. No significant contamination was found in the blanks. All the detected organic compounds were quantified by using the calibration curves established by the authentic standards, with linearities of >0.995.

## 3. Results

### 3.1. Concentrations and Molecular Compositions

#### 3.1.1. n-Alkanes

**Figure 1:**
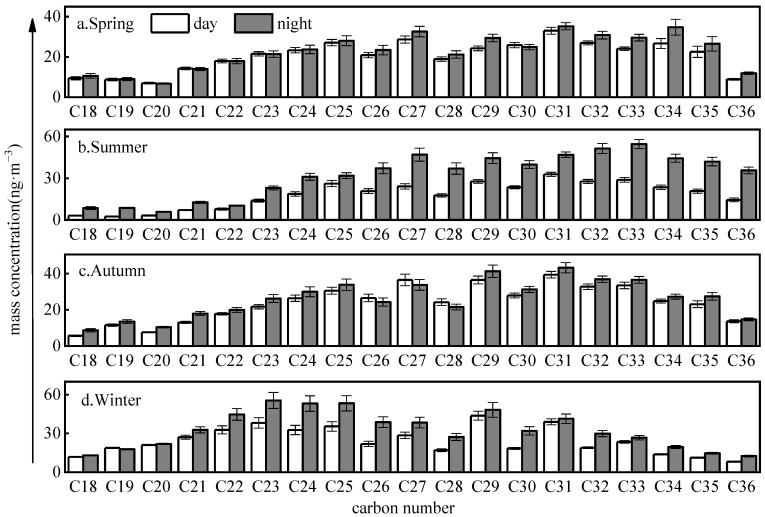
Seasonal and daily variations of n-alkanes mass concentrations (error bars represent min.–max. concentrations).

Table 1 lists the total concentration of 19 homologues (C_18_–C_36_) n-alkanes (Σ_n-alkanes_) in three particle size modes, including fine particle (*Dp* < 2.1 µm), coarse mode (*Dp* > 2.1 µm), and total suspended particle (TSP, *Dp* > 100 µm) in four seasons. The highest average TSP Σ_n-alkanes_ was seen in winter (541 ± 44 ng·m^−3^), followed by summer (478 ± 41 ng·m^−3^), autumn (476 ± 37 ng·m^−3^), and spring (411 ± 34 ng·m^−3^). In terms of daily variability, the Σ_n-alkanes_ concentrations in the nighttime were higher than in the daytime during all seasons (Figure 1). It was found that there was a significant difference between day and night in four seasons with a *p*-value of <0.01 by *t*-test (see the Text Ⅰ for detail), indicating that the relatively lower wind speed and temperature, absence of light, and higher RH at nighttime promote the accumulation of particles in the troposphere of the Guanzhong Plain (Table S1). 

According to the homologue percent profile, there are two main sources of alkanes, which were natural plant wax emission and fossil fuel combustion [21,22]. n-Alkanes from plant waxes usually present the odd carbon number predominance, while those from fossil fuel combustion show no odd/even preference [23]. The carbon preference index (CPI) (the calculation method is shown in Text S1) was usually used to evaluate the emission contributions. CPI of n-alkanes greater than 5 was typically identified as related to plant wax, while that close to 1 was assigned as fossil fuel combustion [8,24]. As shown in Table S2, the daily CPI for TSP show a narrow range from 1.1 ± 0.08 to 1.3 ± 0.17, indicating that fossil fuel combustion was stably predominant in this urban city throughout the year. Previous studies show that the plant waxes consist overall of HMW n-alkanes (i.e., n-C_26_-n-C_36_) [25]. In this study, the proportion of the HMW n-alkanes for TSP presents the highest value in summer (76.4%), in comparison to other seasons (autumn: 69.1%, spring: 67.8%, and winter: 52.2%), that is in accordance with the stronger plant emission in the warmest season. In contrast, the concentrations of low molecular weight (LMW) n-alkanes in winter were 1.7–2.4 times higher than that of other seasons, consistent with the fact that more fossil fuel is burnt in that period. Coal was the most common fuel used for domestic household heating in Baoji and its surrounding rural areas [18]. The plant wax and fossil fuel combustions that contributed to n-alkanes presence could be distinguished by the method described in Text SII. As shown in Table 1, the concentrations of n-alkanes associated with plant wax and fossil fuel ranged from 20.9–101 and 128–441 ng·m^−3^, respectively. These results further confirm the predominant role of the fossil fuel emission with regards to n-alkanes in this city. 

#### 3.1.2. Saccharides

The highest total quantified saccharides for TSP of 651 ± 80.9 ng·m^−3^ was seen in winter, which was 5.4, 8.2, and 4.3 times that in spring (120 ± 22 ng·m^−3^), summer (79.2 ± 6.9 ng·m^−3^), and autumn (151 ± 19 ng·m^−3^), respectively (Table 1). The *p*-value of the *t*-test between winter and other seasons was less than 0.05, which confirms the significant difference in their mass concentrations. Further studies found that the concentrations of Galactosan (Gala), mannosan (Manno), and levoglucosan (Levo) in autumn and winter were 3.7–24.1 times higher than those in spring and summer. To the best of our knowledge, Gala, Manno, and Levo are mainly released from the burning of straws and trees, and thus can be considered as tracers for biomass burning emissions [26,27]. The average contributions of the three compounds to total quantified saccharides were 81.7% and 91.5% in autumn and winter, respectively, much higher than in spring (22.0%) and summer (29.9%). Therefore, biomass burning from surrounding rural areas could be an important contributor to organic aerosols in urban Baoji, particularly during autumn and winter.

**Figure 2:**
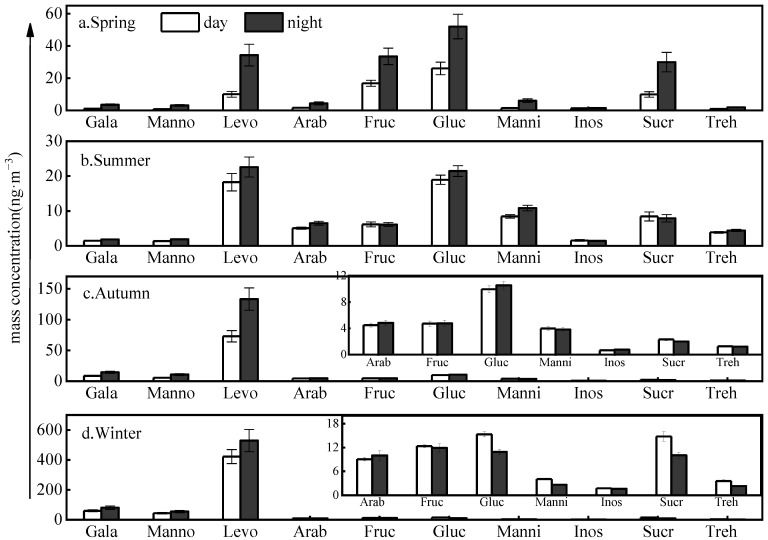
Seasonal and daily variations of carbohydrate mass concentrations (error bars represent min.–max. concentrations).

Non-dehydrated saccharides, including monosaccharides (i.e., glucose (Gluc) and fructose (Fruc)), disaccharides (i.e., sucrose and trehalose), and polyols (i.e., arabitol and mannitol), are mainly emitted by soil microorganisms, plants, and animals [28,29]. In general, the concentrations of the non-dehydrated saccharides in the daytime were higher than at nighttime (Table S2 and Figure 2), consistent with the higher ambient temperature that promotes the metabolism of soil microorganisms and plants. This conclusion was supported by the proportion rate of non-dehydrated saccharides to the total quantified saccharides, presenting the highest value of 77.1% in the summer. Moreover, Gluc and Fruc were mainly released from soil dust and plant debris [30]. The concentration levels of Gluc and Fruc show the highest values in spring, accounting for 32.5% and 21.0% of total quantified saccharides, respectively, probably attributed to the influences of catkins from poplars and willows and emissions from farmland cultivation and vegetation growth [26]. During the flowering season, pollen contributes significantly to sucrose (Sucr) [29,31], explaining the highest concentration and proportion seen in spring. 

#### 3.1.3. PAHs

**Figure 3:**
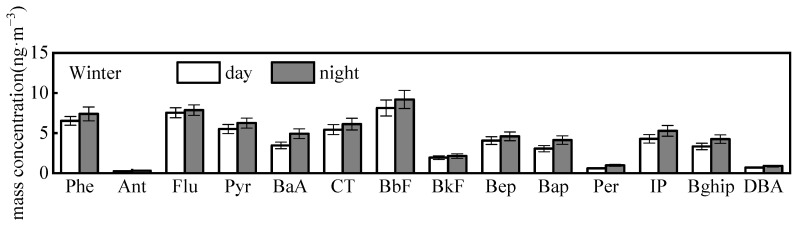
Daily variations of individual PAH mass concentrations in winter (error bars represent min.–max. concentrations).

The abundances of PAHs were usually at least one magnitude lower than n-alkanes and saccharides in the ambient air [2,32]. Due to the use of the impactor multistage sampler for PMs, the amounts of target PAHs distributed into the aerodynamic size fractions were often below MDL during the non-heavy pollution seasons. As result, only the winter concentration levels, and compositions of PAHs could be reported. The average TSP total quantified PAHs was 59.6 ± 6.4 ng·m^−3^ in winter. PAHs in the atmosphere are mainly emitted by the incomplete combustion of fossil fuels and biomass materials [33]. As illustrated in Figure 3, all the individual PAH presents higher concentration at nighttime than that in the daytime. The *t*-test (*p* < 0.01) showed a significant difference between day and night, corresponding to the factors of ambient temperature, photochemical reaction intensity, and atmospheric boundary layer height (BLH). Specifically, the higher daytime temperatures (on average of 6.8 °C) than nighttime (on average of 2.8 °C) result in lower saturated vapor pressure of PAHs, and, as a consequence, the volatilization of semi-volatile PAHs [34]. In addition, sunlight leads the PAHs prone to undergo photochemical decomposition in the daytime [35], while the lower BLH at nighttime was favorable for PAHs and other pollutants accumulations [36].

Among the individual species, “benzo(b) fluoranthene (BbF) and benzo(k)fluoranthene (BkF), which are co-eluted (BbkF) in our GC-system, show the highest contribution to the total quantified PAHs (18.0%), followed by fluoranthene (Flu) (12.9%), and phenanthrene (Phe) (11.7%). Benzo[a]anthracene (BaA), BbF, BkF, benzo[a]pyrene (BaP), indeno[1,2,3-cd]pyrene (InP), and dibenzo[a,e]pyrene (DBA) are well-known carcinogens for humans [35]. The sum concentration of these carcinogens was 24.1 ± 6.4 ng·m^−3^, accounting for 35.7–42.4% of total quantified PAHs. In particular, BaP was characterized as the reference carcinogen, and its average concentration of 3.0–5.1 ng·m^−3^ in 2017, which is 1.2–2.1 times the daily mean concentration limit regulated by the ambient air quality standard (2.5 ng·m^−3^) [37]. The results suggest there is a high potential health risk of PAH exposure for the citizens in this urban city.

### 3.2. Size Distributions

#### 3.2.1. n-Alkanes

**Figure 4:**
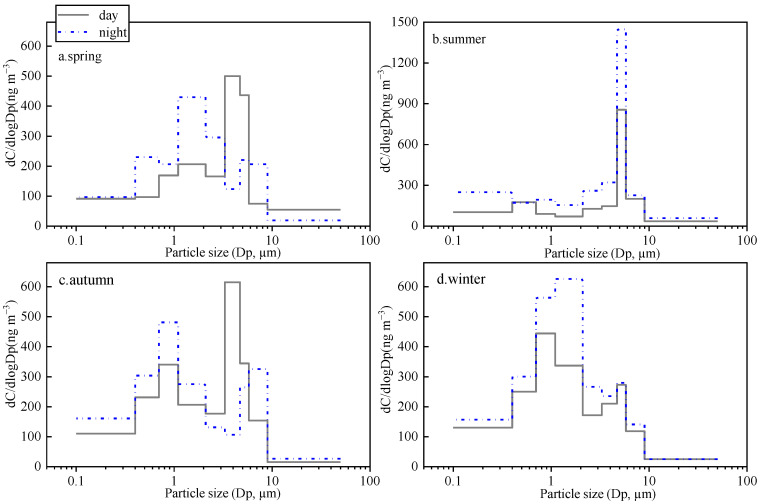
Seasonal particle size distributions of n-alkanes ((**a**). spring day and night; (**b**). summer day and night; (**c**). autumn day and night; (**d**). winter day and night).

Particle size distributions of Σ_n-alkanes_ are illustrated in Figure 4. In general, n-alkanes show a bimodal pattern, presenting two peaks at aerodynamic diameters ranges of 0.7–2.1 and 3.3–4.7 µm in spring, autumn, and winter. In winter, most n-alkanes were distributed in the fine mode of 0.7–2.1 µm both in daytime and nighttime. As shown in Table 1, the concentration of Σ_n-alkanes_ in the fine particles (388 ± 41 ng m^−3^) was 1.5 times higher than that in the coarse mode. Considering that n-alkanes emitted by combustion activities mainly exist in fine particles [38], this distribution again confirms the high contribution of coal combustion and biomass burning for household heating in this urban region. In spring and autumn, the major peak of Σ_n-alkanes_ was found in the fine mode of aerodynamic diameters of 0.7–2.1 µm at nighttime, but it corresponded to the coarse mode of 3.3–4.7 µm in the daytime. This phenomenon could be probably explained by the sowing and harvesting activities during the daytime in the two seasons [39]. In summer, Σ_n-alkanes_ exhibit a unimodal distribution with concentration maxima in coarse mode at aerodynamic diameters of 4.7–5.8 µm in both daytime and nighttime, depending on the strongest plant emissions in the warm season.

#### 3.2.2. Saccharides

**Figure 5:**
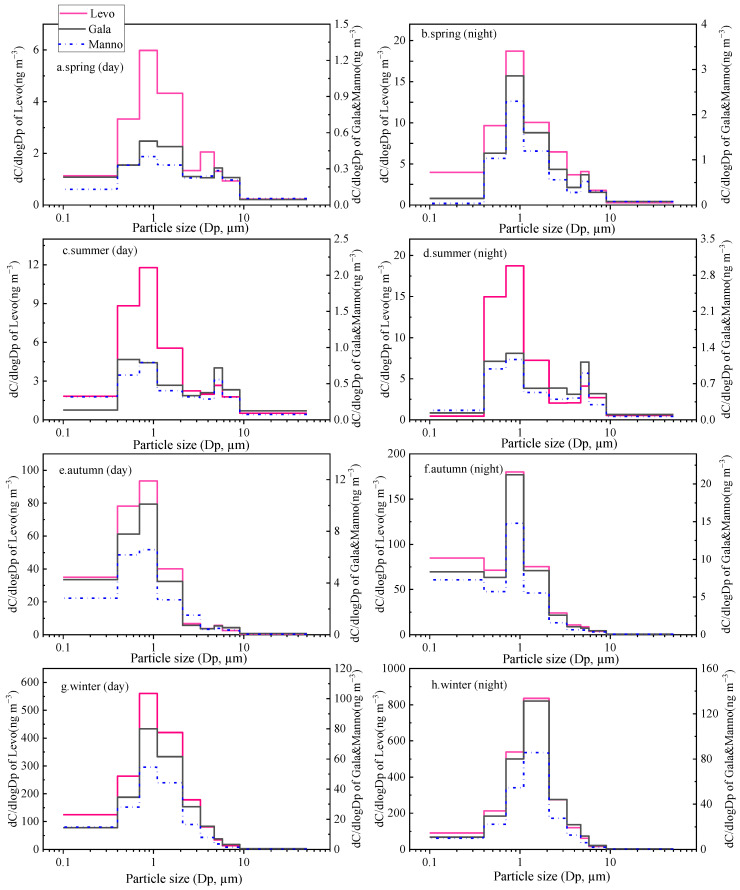
Seasonal particle size distributions of dehydrated anhydrides (**a**). spring day; (**b**). spring night; (**c**). summer day; (**d**). summer night; (**e**). autumn day; (**f**). autumn night; (**g**). winter day; (**h**). winter night.

As shown in Figure 5, the three dehydrated saccharides (i.e., Gala, Manno, and Levo) mostly existed in the fine particles with the maximum corresponding to aerodynamic diameters of 0.7–1.1 μm. This result was consistent with the findings reported in other studies [32,40], which demonstrated that combustion particles have aerodynamic diameters smaller than 2.5 μm (i.e., fine mode). Thus, the size distributions did not display the obvious day–night difference in all seasons. However, in Figure 5c,d, there was a minor peak presented in aerodynamic diameters of 4.7–5.8 μm in spring and summer, probably related to the particle-to-gas partition of the saccharides, and subsequent adsorption onto coarse particles in the warmer season.

**Figure 6:**
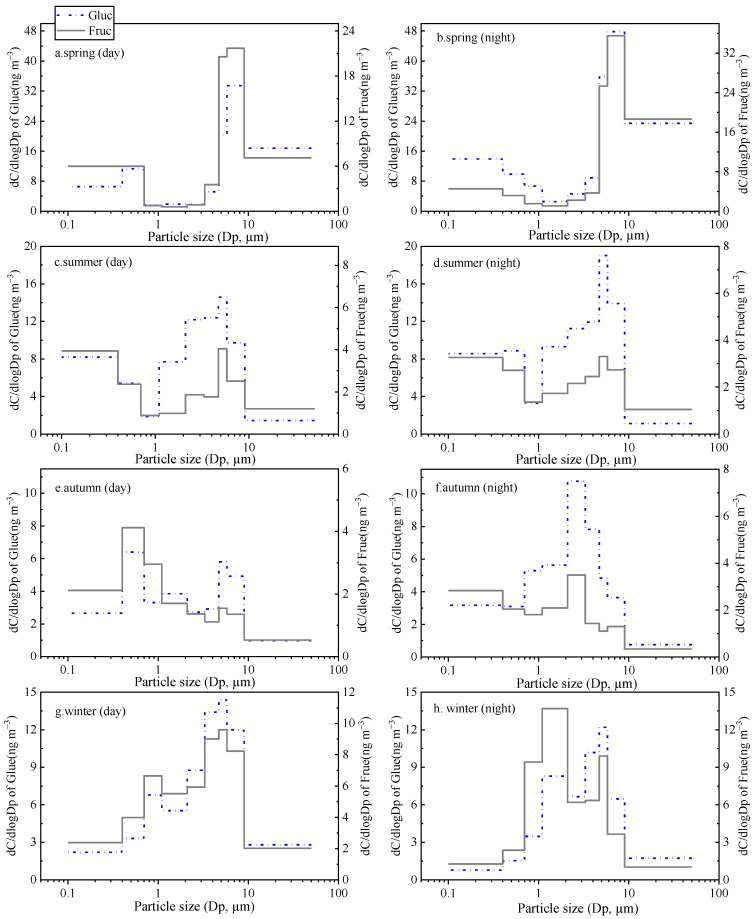
Seasonal particle size distributions of glucose (Gluc) and fructose (Fruc) (**a**). spring day; (**b**).spring night; (**c**). summer day; (**d**). summer night; (**e**). autumn day; (**f**). autumn night; (**g**). winter day; (**h**). winter night.

Glucose (Gluc) and Fructose (Fruc) are the two major metabolites of soil microorganisms, plants, and animals [31,41]. The size distributions of these two saccharides show characteristic seasonal differences (Figure 6). Gluc and Fruc showed a mono-modal size distribution in spring and summer peaking with an aerodynamic diameter of 4.7–9.0 μm in the coarse mode. However, in autumn and winter, two peaks were shown at 0.4–2.1 and 4.7–5.8 μm was shown. In addition, the proportions of the sum of Gluc and Fruc fine particles to TSP in winter (53.7%) and autumn (62.2%) were significantly higher than that in spring (41.9%) and summer (42.2%). These results suggest that the combustion source, such as biomass burning, has a significant contribution to Gluc and Fruc in autumn and winter in the urban area [42,43].

#### 3.2.3. PAHs

**Figure 7:**
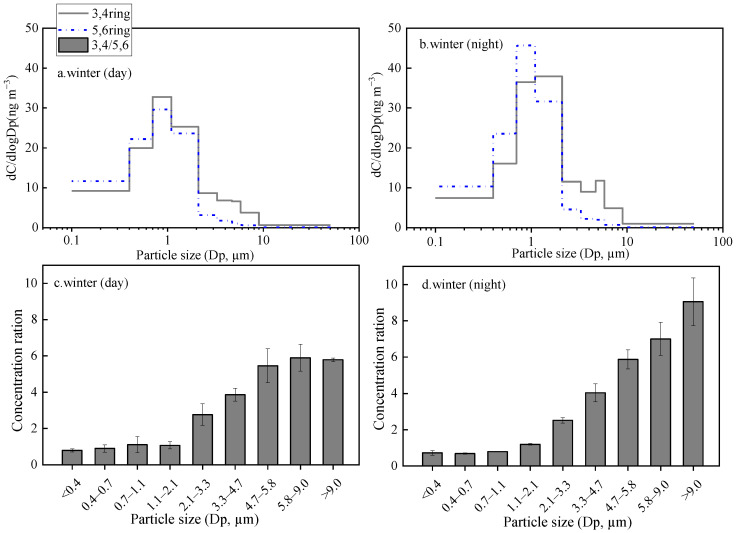
Particle size distributions ((**a**). winter day; (**b**). winter night) and Particle size concentration distributions ((**c**). winter day; (**d**). winter night) of PAHs in winter. (error bars represent min.–max. concentrations).

PAHs are mainly emitted by the incomplete combustion process of carbon-containing matter; thus, they are generally distributed in fine particles [44,45,46]. In this study, the average winter concentration of PAHs in the fine particles (52.4 ± 5.6 ng·m^−3^) is 7.3 times that in the coarse particles (7.2 ± 0.78 ng·m^−3^) (Table 1). The particle size characteristics of both the 3,4-rings and 5,6-rings PAHs show unimodal distributions both in daytime and nighttime, with the maximum at 0.7–2.1 μm (Figure 7). Interestingly, the concentration ratios of 3,4-rings PAHs vs 5,6-ring PAHs increased with the aerodynamic diameters in both daytime and nighttime. The LMW PAHs display semi-volatile properties; hence, they tend to volatilize and move back onto the coarse particles by condensation and/or adsorption [44].

### 3.3. Geometric Mean Diameters (GMD) in the Fine and Coarse Modes

To describe the seasonal and daily differences in size distributions of the target organic compounds, we calculated the average geometric mean diameters (GMD) (the calculation method was provided in Text SⅡ) for the fine (<2.1 µm) and coarse (≥2.1 µm) mode particles (Table 2). The GMDs of all target compounds in the fine mode were larger in winter than those in the spring, summer, and autumn, but an opposite trend was observed for the coarse mode. Since, the mass concentrations of the total n-alkanes and saccharides in winter were 1.1–1.3 and 4.3–8.2 times higher, respectively, than in the other three seasons, and the greater GMD in the fine mode can be partly explained by the enhancement of the coagulation of smaller aerodynamic diameters of the particles in winter. On the other hand, the smaller GMD in the coarse mode could be attributed to back compound condensation accompanying the inversion layer development in winter [40,47]. The mean GMD values of saccharides in the daytime (5.4 ± 3.0 μm) and in the nighttime (3.3 ± 2.1 μm) were higher in coarse mode than fine mode, in contrast to those in summer, autumn, and winter which were more concentrated in the fine mode particles between 0.72 ± 0.08 and 1.8 ± 0.21 μm, respectively.

The average GMD of winter PAHs on the fine mode in daytime and nighttime are 0.67 ± 0.17 μm and 0.79 ± 0.04 μm, respectively. The values were lower than that of Mt. Hua (0.90 ± 0.02 μm) [32] and Mt. Tai (0.99 ± 0.10 μm) [19] in wintertime. In the urban areas, PAHs were mainly from fossil fuel burning, but in alpine areas, they were mainly from biomass burning. This latter releases larger particles than fossil fuel combustion [48]. Compared to HMW PAHs (5,6-rings), LMW PAHs (3,4-rings) exhibit greater GMD due to the particle redistribution effect [49].

### 3.4. Comparison of n-Alkanes and PAHs to 2008: Implication for the Achievement of Pollution Reduction in the Recent Decade

According to the data of the Baoji Municipal Bureau of Statistics, the energy structure and vehicle ownership varied significantly accompanying pollution control and economic growth in the recent decade. For example, the proportion of secondary industry in 2017 (64.5%) [50] slightly increases in comparison with that in 2008 (60.3%) [51], but industrial coal consumption significantly decreases from 8.9 million tons in 2008 [52] to 5.8 million tons in 2017 [53]. Vehicle ownership in 2017 was 322,000 [50], nearly three times that in 2008 (104,000) [51]. Additionally, the total consumption of coal and natural gas in Shaanxi province in 2017 increased by ~81% and ~96%, respectively, compared with 2008, and the consumption of crude oil decreased by ca.21% (Figure S2). These results suggested that energy emission still presents an increasing trend in recent years, but air quality indeed improved due to the control strategies in Baoji.

Fortunately, Wang et al. [19] determined the concentrations and particle size distributions of n-alkanes and PAHs in Baoji in the spring and winter of 2008. The sampling time was performed on 11–14 January and 12–20 February 2008 in winter and 12–24 April 2008 in spring. The sampling sites studied in this study and the previous study were both located in urban areas, and the distance between the two sampling sites is only about 6 km. The sampling protocols and frequencies between the two studies were comparable, providing us with a reference to access the beneficial impact of abating the organic aerosols in the city. Table 3 summarizes the concentration levels and diagnostic ratios of n-alkanes and PAHs in the spring and winter of 2008 and 2017. In spring, the mass concentrations of the fossil fuel-derived n-alkanes decreased from 413 ± 124 ng·m^−3^ in 2008 to 362 ± 16.6 ng·m^−3^ in 2017. The reductions were more significant for the wintertime, from 1470 ± 482 ng·m^−3^ in 2008 to nearly one-third of 440 ± 30 ng·m^−3^ in 2017. Similarly, the concentrations of PAHs in winter also decrease from 161 ± 39 ng·m^−3^ in 2008 to 59.6 ± 6.43 ng·m^−3^ in 2017. These results prove that the pollution control measures conducted by the local government in recent years could improve the air quality in Baoji city. Table 3 compares the diagnostic ratios of PAHs between the two years. The reference diagnostic ratios of BghiP/BeP (0.80) and IP/BghiP (1.26) can be used to characterize coal burning [33,54]. The BghiP/BeP and IP/BghiP ratios were 1.2 ± 0.0 and 1.2 ± 0.1, respectively, in 2008, in comparison to 0.87 ± 0.10 and 1.3 ± 0.07, in 2017. These values suggest that coal combustion is still an important contributor to PAHs. Moreover, the variation of the diagnostic ratios of Ant/(Ant + Phe) supports the increase in the contribution of vehicle emissions from 2008 to 2017 [55].

**Figure 8:**
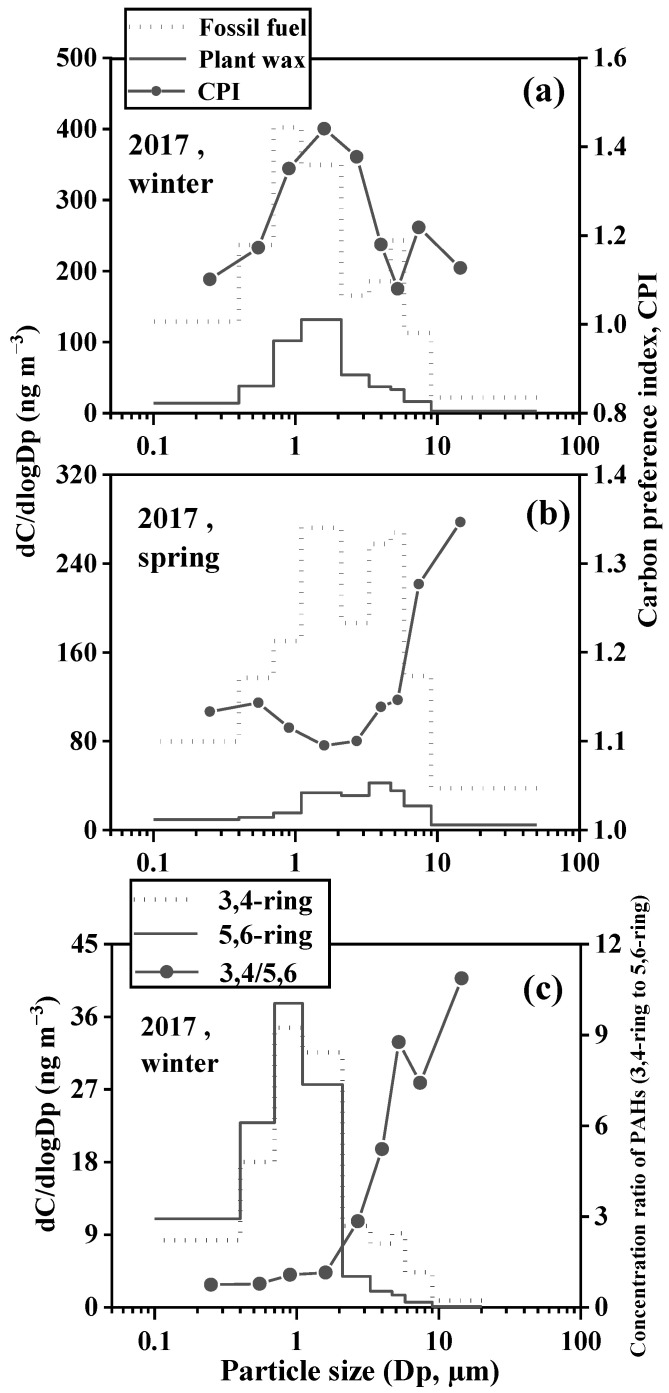
Particle size distributions of fossil fuel- and plant wax-derived n-alkanes and CPI values PAHs and CPI values in Baoji ((**a**). winter 2017; (**b**). spring 2017; (**c**). winter 2017).

Size distributions of the plant wax and fossil fuel-derived n-alkanes and 3/4 and 5/6-rings PAHs in the spring and winter of 2017 were illustrated in Figure 8. The concentration of the n-alkanes coming from fossil fuels was much higher than that of the plant waxes, confirming that fossil fuel emission was a dominant source of n-alkanes. The size distribution of n-alkanes was mono-modal in spring and winter in 2008, peaking at 0.7–2.1 µm [19]. However, in 2017, a bimodal pattern was seen with a major peak at 0.7–2.1 µm and a minor one at 4.7–5.8 µm. The plant wax-derived n-alkanes show a unimodal distribution in both 2008 and 2017. The trends of the CPI values were similar between the two years which were close to unity for the finest particles (<0.4 µm) and raise with the particle sizes in spring. This supports the elaboration of the predominance of the fossil fuel-derived n-alkanes on the finest size particles, whereas the input from the plant wax becomes more significant with high particle sizes in spring [19]. The decrease in CPI with the coarse fraction in the winter aerosols was mostly due to relatively lower abundances of biogenic n-alkanes, which easily settled with large particles under stagnant conditions [19].

PAHs peaked at aerodynamic diameters of 0.7 µm < *Dp* < 1.1 µm in both 2008 and 2017, in agreement with their accumulation in fine particles [44,45,46]. In 2017, the 3,4-rings PAHs show a minor peak with aerodynamic diameters of 4.7 µm < *Dp* < 5.8 µm, probably due to the slightly higher average ambient temperature (4.8 °C) than that in 2008 (0.32 °C) (Table S1). The higher temperatures were favorable for the adsorption of relatively volatile 3,4-ring PAHs on the coarse particles. Meanwhile, the relative abundance of 3,4-rings PAHs to the 5,6-ring PAHs was lower in finer size particles but higher in larger particles. This could be ascribed to the LMW PAHs in the particulate phase could evaporate into the atmosphere and subsequently adsorb/condense onto pre-existing particles [56].

## 4. Conclusions

In Baoji city, the concentrations of both n-alkanes and saccharides were maximum in winter, i.e., 1.1–1.3 and 4.3–8.2 times, respectively, those in spring, summer, and autumn. The consistent CPI values of n-alkanes indicate the stable contributions of fossil fuels throughout the year. n-Alkanes were mainly distributed in fine particles in spring, autumn, and winter, but in coarse particles in summer. The proportions of dehydrated sugar were much higher in autumn and winter, consistent with the dominance of biomass burning in the heating seasons. The concentrations of non-dehydrated saccharides and all individual PAH present higher concentrations at nighttime than the daytime, ascribed to the lower temperature, photochemical reaction intensity, and BLH. Unfortunately, the average concentration of BaP in 2017 still exceeds the daily concentration set by the ambient air quality standard. Dehydrated saccharides and PAHs mostly exist in fine particles. GMDs for all the target compounds in the fine mode were larger in winter than in the other three seasons, whereas the reverse was observed in the coarse mode. Compared with 2008, the mass concentration of fossil fuel-derived n-alkane and PAHs decreased significantly in 2017, indicating that the control strategies implemented conducted by the local government could mitigate air pollution. Even though coal combustion has been significantly controlled in recent years, the diagnostic ratios of PAHs indicate that their contribution cannot be ignored in the heating seasons. Meanwhile, the controls of vehicle emissions could not sufficiently offset the impact of gentle increases in ownership of fossil-fueled motor vehicles. It was recommended to promote and replace old vehicles with eco-friendly and energy-saving models.

## Figures and Tables

**Table 1:** Mass concentrations of n-alkanes, saccharides, and PAHs (ng·m^−3^) of the fine (<2.1 µm) and coarse (≥2.1 µm) particles and the whole range of impactor particle sizes (total) in the four seasons.

Index	Spring			Summer			Autumn			Winter		
	TSP	Fine	Coarse	TSP	Fine	Coarse	TSP	Fine	Coarse	TSP	Fine	Coarse
n-Alkanes	411 ± 35	223 ± 34	189 ± 28	478 ± 41	208 ± 39	270 ± 38	476 ± 37	295 ± 22	181 ± 31	541 ± 44	387 ± 41	154 ± 9
Plant wax	49.5 ± 5.3	22.6 ± 4.5	27.0 ± 5.3	55.1 ± 4.7	20.9 ± 4.5	34.2 ± 4.4	65.9 ± 6.8	40.3 ± 3.2	25.4 ± 6.7	101 ± 12	75.0 ± 15.1	25.8 ± 3.3
Fossil fuel	362 ± 30	200 ± 31	162 ± 23	423 ± 36	187 ± 35	236 ± 34	410 ± 31	254 ± 20	156 ± 25	441 ± 33	312 ± 28	128 ± 6
CPI	1.2 ± 0.14	1.1 ± 0.09	1.20 ± 0.15	1.15 ± 0.09	1.13 ± 0.07	1.16 ± 0.11	1.19 ± 0.13	1.23 ± 0.08	1.16 ± 0.14	1.23 ± 0.21	1.27 ± 0.22	1.20 ± 0.19
Saccharides	120 ± 22	67.1 ± 22.4	53.0 ± 12.5	79.2 ± 6.9	44.9 ± 9.0	34.3 ± 4.3	151 ± 19.1	131 ± 18	19.8 ± 2.7	651 ± 81	530 ± 87	121 ± 23
Dehydrated	26.4 ± 5.4	23.8 ± 7.2	2.61 ± 0.29	23.7 ± 3.2	18.7 ± 3.7	5.0 ± 0.90	123 ± 17.7	116 ± 16	7.5 ± 1.5	595 ± 80	508 ± 83	87.6 ± 22.6
Non-dehydrated	93.7 ± 18.0	43.3 ± 16.0	50.4 ± 12.6	55.5 ± 4.6	26.2 ± 5.9	29.3 ± 3.8	27.7 ± 2.1	15.4 ± 2.4	12.3 ± 1.5	55.2 ± 3.3	21.5 ± 4.3	33.7 ± 1.9
PAHs										59.6 ± 7.1	52.4 ± 5.6	7.2 ± 0.8
3,4-ring	-^	-	-	-	-	-	-	-	-	30.8 ± 3.3	25.1 ± 3.0	5.7 ± 0.5
5,6-ring	-	-	-	-	-	-	-	-	-	28.8 ± 3.9	27.3 ± 2.7	1.4 ± 0.3

^ Concentrations in the impact samples of the three seasons are below the detection limit.

**Table 2:** Geometric mean diameter (GMD, µm) of the fine (<2.1 µm) and coarse (≥2.1 µm) particles and the whole range of particle sizes (total) in the four seasons (the confidence interval represents the standard deviation).

	Spring			Summer			Autumn			Winter		
	Fine	Coarse	Total	Fine	Coarse	Total	Fine	Coarse	Total	Fine	Coarse	Total
***I. Daytime***												
Σn-alkanes	0.69 ± 0.15	8.8 ± 2.8	2.7 ± 1.2	0.48 ± 0.02	8.2 ± 2.5	2.4 ± 0.64	0.66 ± 0.10	5.5 ± 1.5	1.7 ± 0.40	0.70 ± 0.16	6.7 ± 1.0	1.4 ± 0.37
Fossil fuel	0.69 ± 0.15	8.9 ± 2.8	2.6 ± 1.2	0.48 ± 0.03	8.1 ± 2.5	2.4 ± 0.67	0.67 ± 0.10	5.5 ± 1.5	1.7 ± 0.42	0.67 ± 1.45	6.9 ± 1.1	1.4 ± 0.38
Plant wax	0.73 ± 0.13	9.1 ± 3.2	3.3 ± 0.18	0.47 ± 0.02	8.9 ± 2.4	2.4 ± 0.37	0.63 ± 0.10	5.6 ± 2.2	1.7 ± 0.34	0.83 ± 0.21	5.8 ± 0.70	1.4 ± 0.30
Saccharides	0.45 ± 0.08	21.4 ± 4.0	5.4 ± 3.01	0.44 ± 0.01	9.2 ± 1.5	1.8 ± 0.21	0.60 ± 0.11	7.8 ± 1.5	0.89 ± 0.20	0.76 ± 0.19	5.0 ± 0.14	1.1 ± 0.17
PAHs										0.67 ± 0.17	5.5 ± 0.23	0.85 ± 0.18
3,4-ring	-^	-	-	-	-	-	-	-	-	0.71 ± 0.17	5.8 ± 0.30	1.01 ± 0.18
5,6-ring	-	-	-	-	-	-	-	-	-	0.64 ± 0.16	4.3 ± 0.54	0.70 ± 0.19
***II. Nighttime***												
Σn-alkanes	0.79 ± 0.07	6.5 ± 2.0	1.7 ± 0.32	0.46 ± 0.02	8.1 ± 1.4	1.9 ± 0.45	0.64 ± 0.03	8.1 ± 0.85	1.4 ± 0.08	0.80 ± 0.03	6.1 ± 0.28	1.4 ± 0.13
Fossil fuel	0.79 ± 0.04	6.4 ± 2.0	1.7 ± 0.34	0.46 ± 0.02	8.2 ± 1.6	1.8 ± 0.41	0.64 ± 0.04	8.1 ± 0.90	1.4 ± 0.08	0.74 ± 0.03	6.4 ± 0.19	1.3 ± 0.17
Plant wax	0.77 ± 0.32	7.6 ± 2.9	1.9 ± 0.60	0.43 ± 0.05	7.6 ± 1.7	2.1 ± 0.77	0.65 ± 0.02	7.9 ± 0.55	1.4 ± 0.44	1.06 ± 0.04	4.9 ± 0.25	1.6 ± 0.13
Saccharides	0.41 ± 0.19	18.7 ± 5.5	3.3 ± 2.10	0.51 ± 0.16	7.7 ± 3.9	1.5 ± 0.55	0.55 ± 0.05	5.4 ± 0.48	0.7 ± 0.08	1.00 ± 0.04	4.0 ± 0.05	1.3 ± 0.14
PAHs										0.79 ± 0.04	5.6 ± 0.42	1.0 ± 0.08
3,4-ring	-	-	-	-	-	-	-	-	-	0.85 ± 0.06	6.0 ± 0.45	1.3 ± 0.15
5,6-ring	-	-	-	-	-	-	-	-	-	0.74 ± 0.04	4.2 ± 0.27	0.81 ± 0.04
***III. Average***												
Σn-alkanes	0.74 ± 0.12	7.7 ± 2.6	2.2 ± 0.9	0.47 ± 0.02	8.2 ± 1.8	2.2 ± 0.58	0.65 ± 0.07	6.8 ± 1.8	1.6 ± 0.31	0.75 ± 0.11	6.4 ± 0.74	1.4 ± 0.25
Fossil fuel	0.74 ± 0.11	7.6 ± 2.6	2.1 ± 1.0	0.47 ± 0.02	8.2 ± 1.9	2.1 ± 0.59	0.65 ± 0.07	6.8 ± 1.8	1.6 ± 0.32	0.71 ± 0.10	6.7 ± 0.74	1.4 ± 0.27
Plant wax	0.75 ± 0.22	8.4 ± 2.9	2.6 ± 0.9	0.45 ± 0.04	8.2 ± 2.0	2.2 ± 0.56	0.64 ± 0.07	6.8 ± 1.9	1.5 ± 0.39	0.95 ± 1.84	5.3 ± 0.67	1.5 ± 0.22
Saccharides	0.43 ± 0.13	20.0 ± 4.5	4.4 ± 3.7	0.48 ± 0.11	8.4 ± 2.7	1.7 ± 0.41	0.57 ± 0.08	6.6 ± 1.6	0.80 ± 0.16	0.88 ± 0.18	4.5 ± 0.52	1.2 ± 0.19
PAHs										0.73 ± 0.13	5.5 ± 0.30	0.94 ± 0.16
3,4-ring	-	-	-	-	-	-	-	-	-	0.78 ± 0.14	5.9 ± 0.36	1.1 ± 0.21
5,6-ring	-	-	-	-	-	-	-	-	-	0.69 ± 0.12	4.3 ± 0.39	0.76 ± 0.14

^ Concentrations in the impact samples of the three seasons were below the detection limit.

**Table 3:** Mass concentration of organic compounds and the diagnostic ratio of PAHs in Baoji in 2017 and 2008 (the confidence interval represents the standard deviation).

	2017				2008 ^#^	
	Spring	Summer	Autumn	Winter	Spring	Winter
n-Alkanes						
∑n-alkane	411 ± 19	478 ± 31	476 ± 21	541 ± 39	1698 ± 568	487 ± 145
Plant wax *	49.5 ± 2.5	55.1 ± 3.6	65.9 ± 3.7	101 ± 11	74 ± 21	228 ± 87
Fossil fuel **	362 ± 17	423 ± 27	410 ± 18	441 ± 30	413 ± 124	1470 ± 482
CPI	1.2 ± 0.10	1.15 ± 0.07	1.2 ± 0.10	1.2 ± 0.17	1.3 ± 0.0	1.2 ± 0.0
PAHs						
ΣPAHs	-^	-	-	59.6 ± 6.4	536 ± 80	161 ± 39
BeP/(BeP + BaP)	-	-	-	0.55 ± 0.03	0.47 ± 0.0	0.47 ± 0.0
IP/BghiP	-	-	-	1.26 ± 0.07	1.1 ± 0.3	1.2 ± 0.1
BghiP/BeP	-	-	-	0.87 ± 0.10	1.5 ± 0.1	1.2 ± 0.0
Ant/(Ant + Phe)	-	-	-	0.04 ± 0.01	0.11 ± 0.0	0.22 ± 0.0

# The data achieved by Wang et al. [19]. * Plant wax is calculated as the excess odd homologues-adjacent even homologues average [25]. ** The difference from the total n-alkanes is the fossil fuel-derived amount [25]. ^ Concentrations in the impact samples of the three seasons were below the detection limit.

## Data Availability

Data used in this study are available by request from the corresponding author.

## Supplementary Materials

The following supporting information can be downloaded at: https://www.mdpi.com/article/10.3390/toxics11020164/s1, Table S1: Meteorological parameters in Baoji city during the sampling periods; Table S2: Diurnal concentrations of n-alkanes, PAHs, and saccharides in the four seasons (ng·m^−3^) (The confidence interval represents the standard deviation); Figure S1: Interannual variation of SO_2_, NO_2_, and PM10 mass concentrations in Baoji city from 2008 to 2017; Figure S2: Interannual variation of energy consumption in Shaanxi Province [57].

## References

[1] An Z.S., Huang R.J., Zhang R.Y., Tie X.X., Li G.H., Cao J.J., Zhou W.J., Shi Z.G., Han Y.M., Gu Z.L. (2019). Severe haze in northern China: A synergy of anthropogenic emissions and atmospheric processes. Proc. Natl. Acad. Sci. USA.

[2] Li J.J., Wang G.H., Zhang Q., Li J., Wu C., Jiang W.Q., Zhu T., Zeng L.M. (2019). Molecular characteristics and diurnal variations of organic aerosols at a rural site in the North China Plain with implications for the influence of regional biomass burning. Atmos. Chem. Phys..

[3] Tremblay R.T., Riemer D.D., Zika R.G. (2007). Organic composition of PM_2.5_ and size-segregated aerosols and their sources during the 2002 Bay Regional Atmospheric Chemistry Experiment (BRACE), Florida, USA. Atmos. Environ..

[4] Ladji R., Yassaa N., Balducci C., Cecinato A. (2014). Particle size distribution of n-alkanes and polycyclic aromatic hydrocarbons (PAHS) in urban and industrial aerosol of Algiers, Algeria. Environ. Sci. Pollut. Res. Int..

[5] Jie K.M., Jie R.L., Hong R., Ye Z., Kimitaka K., Liang Z.H., Fang W.L., Le S.Y., Fa W.Z., Qing F.P. (2018). Primary biogenic and anthropogenic sources of organic aerosols in Beijing, China: Insights from saccharides and n-alkanes. Environ. Pollut..

[6] Nemmar A., Hoet P.H.M., Vanquickenborne B., Dinsdale D., Thomeer M., Hoylaerts M.F., Vanbilloen H., Mortelmans L., Nemery B. (2002). Passage of inhaled particles into the blood circulation in humans. Circulation.

[7] Wang G.H., Kawamura K., Lee S.C., Ho K.F., Cao J.J. (2006). Molecular, seasonal, and spatial distributions of organic aerosols from fourteen Chinese cities. Environ. Sci. Technol..

[8] Kang M., Kim K., Choi N., Kim Y.P., Lee J.Y. (2020). Recent Occurrence of PAHs and n-Alkanes in PM_2.5_ in Seoul, Korea and Characteristics of Their Sources and Toxicity. Int. J. Environ. Res. Public Health.

[9] Ames B.N., McCann J., Yamasaki E. (1975). Methods for detecting carcinogens and mutagens with the salmonella/mammalianmicrosome mutagenicity test. Mutat. Res..

[10] Alves C., Vicente A., Pio C., Kiss G., Hoffer A., Decesari S., Prevot A.S.H., Cruz Minguillon M., Querol X., Hillamo R. (2012). Organic compounds in aerosols from selected European sites-Biogenic versus anthropogenic sources. Atmos. Environ..

[11] Kong S., Ding X., Bai Z., Han B., Chen L., Shi J.W., Li Z. (2010). A seasonal study of polycyclic aromatic hydrocarbons in PM_2.5_ and PM_2.5–10_ in five typical cities of Liaoning Province, China. J. Hazard. Mater..

[12] Yang T.T., Hsu C.Y., Chen Y.C., Young L.H., Huang C.H., Ku C.H. (2017). Characteristics, Sources, and Health Risks of Atmospheric PM_2.5_-Bound Polycyclic Aromatic Hydrocarbons in Hsinchu, Taiwan. Aerosol Air Qual. Res..

[13] Dat N.-D., Chang M.B. (2017). Review on characteristics of PAHs in atmosphere, anthropogenic sources and control technologies. Sci. Total Environ..

[14] Li J.J., Li J., Wang G.H., Ho K.F., Han J., Dai W.T., Wu C., Cao C., Liu L. (2022). In-vitro oxidative potential and inflammatory response of ambient PM_2.5_ in a rural region of Northwest China: Association with chemical compositions and source contribution. Environ. Res..

[15] Fu P., Zhuang G., Sun Y., Wang Q., Chen J., Ren L., Yang F., Wang Z., Pan X., Li X. (2016). Molecular markers of biomass burning, fungal spores and biogenic SOA in the Taklimakan desert aerosols. Atmos. Environ..

[16] Wang Y.C., Li X., Wang Q.Y., Zhou B.H., Liu S.X., Tian J., Hao Q., Li G.H., Han Y.M., Ho S.S.H. (2022). Response of aerosol composition to the clean air actions in Baoji city of Fen-Wei River Basin. Environ. Res..

[17] Wang G., Xie M., Hu S., Gao S., Tachibana E., Kawamura K. (2010). Dicarboxylic acids, metals and isotopic compositions of C and N in atmospheric aerosols from inland China: Implications for dust and coal burning emission and secondary aerosol formation. Atmos. Chem. Phys..

[18] Xie M.J., Wang G.H., Hu S.Y., Han Q.Y., Xu Y.J., Gao Z.C. (2009). Aliphatic alkanes and polycyclic aromatic hydrocarbons in atmospheric PM10 aerosols from Baoji, China: Implications for coal burning. Atmos. Res..

[19] Wang G.H., Kawamura K., Xie M.J., Hu S.Y., Gao S., Cao J.J., An Z.S., Wang Z. (2009). Size-distributions of n-alkanes, PAHs and hopanes and their sources in the urban, mountain and marine atmospheres over East Asia. Atmos. Chem. Phys..

[20] Wang G.H., Kawamura K. (2005). Molecular characteristics of urban organic aerosols from Nanjing: A case study of A mega-city in China. Environ. Sci. Technol..

[21] Brown S.G., Herckes P., Ashbaugh L., Hannigan M.P., Kreidenweis S.M., Collett J.L. (2002). Characterization of organic aerosol in Big Bend National Park, Texas. Atmos. Environ..

[22] Sun N., Li X., Ji Y., Huang H., Ye Z., Zhao Z. (2021). Sources of PM_2.5_-Associated PAHs and n-alkanes in Changzhou China. Atmosphere.

[23] Li J.J., Wang G.H., Wang X.M., Cao J.J., Sun T., Cheng C.L., Meng J.J., Hu T.F., Liu S.X. (2013). Abundance, composition and source of atmospheric PM2.5 at a remote site in the Tibetan Plateau, China. Tellus B Chem. Phys. Meteorol..

[24] Hernandez-Guzman F.A., Macias-Zamora J.V., Ramirez-alvarez N., Quezada-Hernandez C., Ortiz-Lopez R. (2021). Source identification of n-alkanes and isoprenoids using diagnostic ratios and carbon isotopic composition on crude oils and surface waters from the Gulf of Mexico. Environ. Monit. Assess..

[25] Simoneit B.R.T., Elias V.O., Kobayashi M., Kawamura K., Rushdi A.I., Medeiros P.M., Rogge W.F., Didyk B.M. (2004). Composition and major sources of organic compounds of aerosol particulate matter sampled during the ACE-Asia campaign. J. Geophys. Res.-Atmos..

[26] Liang L., Engling G., Du Z., Cheng Y., Duan F., Liu X., He K. (2016). Seasonal variations and source estimation of saccharides in atmospheric particulate matter in Beijing, China. Chemosphere.

[27] Bhattarai H., Saikawa E., Wan X., Zhu H.X., Ram K., Gao S.P., Kang S.C., Zhang Q.G., Zhang Y.L., Wu G.M. (2019). Levoglucosan as a tracer of biomass burning: Recent progress and perspectives. Atmos. Res..

[28] Elbert W., Taylor P.E., Andreae M.O., Poschl U. (2007). Contribution of fungi to primary biogenic aerosols in the atmosphere: Wet and dry discharged spores, carbohydrates, and inorganic ions. Atmos. Chem. Phys..

[29] Fu P.Q., Kawamura K., Kobayashi M., Simoneit B.R.T. (2012). Seasonal variations of sugars in atmospheric particulate matter from Gosan, Jeju Island: Significant contributions of airborne pollen and Asian dust in spring. Atmos. Environ..

[30] Simoneit B.R.T., Elias V.O., Kobayashi M., Kawamura K., Rushdi A.I., Medeiros P.M., Rogge W.F., Didy B.M. (2004). Sugars dominant Water-Soluble Organic Compounds in Soils and Characterization as Tracers in Atmospheric Particulate Matter. Environ. Sci. Technol..

[31] Haque M.M., Verma S.K., Deshmukh D.K., Kunwar B., Miyazaki Y., Kawamura K. (2021). Seasonal and temporal variations of ambient aerosols in a deciduous broadleaf forest from northern Japan: Contributions of biomass burning and biological particles. Chemosphere.

[32] Li J.J., Wang G.H., Zhou B.H., Cheng C.L., Cao J.J., Shen Z.X., An Z.S. (2012). Airborne particulate organics at the summit (2060 m, a.s.l.) of Mt. Hua in central China during winter: Implications for biofuel and coal combustion. Atmos. Res..

[33] Ravindra K., Sokhi R., Vangrieken R. (2008). Atmospheric polycyclic aromatic hydrocarbons: Source attribution, emission factors and regulation. Atmos. Environ..

[34] Li J.J., Wang G.H., Aggarwal S.G., Huang Y., Ren Y.Q., Zhou B.H., Singh K., Gupta P.K., Cao J.J., Zhang R. (2014). Comparison of abundances, compositions and sources of elements, inorganic ions and organic compounds in atmospheric aerosols from Xi’an and New Delhi, two megacities in China and India. Sci. Total Environ..

[35] Singh D.K., Kawamura K., Yanase A., Barrie L.A. (2017). Distributions of Polycyclic Aromatic Hydrocarbons, Aromatic Ketones, Carboxylic Acids and Trace Metals in Arctic Aerosols: Long-Range Atmospheric Transport and Photochemical Degradation/Production at Polar Sunrise. Environ. Sci. Technol..

[36] Liu X.D., Meng J.J., Hou Z.F., Yan L., Wang G.H., Yi Y.N., Wei B.J., Fu M.X., Li J.J., Cao J.J. (2019). Molecular Compositions and Sources of Organic Aerosols from Urban Atmosphere in the North China Plain during the Wintertime of 2017. Aerosol Air Qual. Res..

[37] Liu X.D., Hou Z.F., Meng J.J., Yan L., Zhou B.B., Liu Z.T., Yi Y.N., Li J. (2019). Source analysis and health risk assessment of PAHs in PM_2.5_ in Liaocheng city in winter. Environ. Sci..

[38] Bi X.H., Sheng G.Y., Peng P.A., Chen Y.J., Fu J.M. (2005). Size distribution of n-alkanes and polycyclic aromatic hydrocarbons (PAHs) in urban and rural atmospheres of Guangzhou, China. Atmos. Environ..

[39] Kavouras I.G., Stephanou E.G. (2002). Particle size distribution of organic primary and secondary aerosol constituents in urban, background marine, and forest atmosphere. J. Geophys. Res.-Atmos..

[40] Wang G.H., Kawamura K., Xie M.J., Hu S.Y., Li J.J., Zhou B.H., Cao J.J., An Z.S. (2011). Selected water-soluble organic compounds found in size-resolved aerosols collected from urban, mountain and marine atmospheres over East Asia. Tellus B.

[41] Caseiro A., Marr I.L., Claeys M., Kasper-Giebl A., Puxbaum H., Pio C.A. (2007). Determination of saccharides in atmospheric aerosol using anion-exchange high-performance liquid chromatography and pulsed-amperometric detection. J. Chromatogr. A.

[42] Oduber F., Calvo A.I., Castro A., Alves C., Blanco-Alegre C., Fernandez-Gonzalez D., Barata J., Calzolai G., Nava S., Lucarelli F. (2021). One-year study of airborne sugar compounds: Cross-interpretation with other chemical species and meteorological conditions. Atmos. Res..

[43] Alves C.A., Vicente A., Monteiro C., Gonçalves C., Evtyugina M., Pio C. (2011). Emission of trace gases and organic components in smoke particles from a wildfire in a mixed-evergreen forest in Portugal. Sci. Total Environ..

[44] Ren Y., Zhou B., Tao J., Cao J., Zhang Z., Wu C., Wang J., Li J., Zhang L., Han Y. (2017). Composition and size distribution of airborne particulate PAHs and oxygenated PAHs in two Chinese megacities. Atmos. Res..

[45] Yin H., Xu L. (2018). Comparative study of PM10/PM2.5-bound PAHs in downtown Beijing, China: Concentrations, sources, and health risks. J. Clean. Prod..

[46] Song W.H., Cao F., Lin Y.C., Haque M.M., Wu X., Zhang Y.X., Zhang C.Y., Xie F., Zhang Y.L. (2021). Extremely high abundance of polycyclic aromatic hydrocarbons in aerosols from a typical coal-combustion rural site in China: Size distribution, source identification and cancer risk assessment. Atmos. Res..

[47] Herner J.D., Ying Q., Aw J., Gao O., Chang D.P.Y., Kleeman M.J. (2006). Dominant Mechanisms that Shape the Airborne Particle Size and Composition Distribution in Central California. Aerosol Sci. Technol..

[48] Wang G.H., Kawamura K., Xie M.J., Hu S.Y., Cao J., An Z.S., Waston J.G., Chow J.C. (2009). Organic molecular compositions and size distributions of chinese summer and autumn aerosols from nanjing: Characteristic haze event caused by wheat straw burning. Environ. Sci. Technol..

[49] Yan L., Xiang L. (2020). Volatility Dependence of the Aerosol Size Distributions of Nonpolar Organic Compounds: A Case Study in Shanghai. J. Geophys. Res.-Atmos..

[50] Baoji-Statistics-Bureau (2017). 2017 Baoji National Economic and Social Development Statistical Bulletin.

[51] Baoji-Statistics-Bureau (2008). 2008 Baoji National Economic and Social Development Statistical Bulletin.

[52] Master-plan-of-Baoji (2008). Baoji City General Planning Eia Water, Energy.

[53] Shaan-xi-Provincial-Bureau-of-Statistics (2018). Shaanxi Statistical Yearbook.

[54] Takeshi O., Takashi A., Masahiro F., Hidetsuru M. (2004). Polycyclic aromatic hydrocarbons in indoor and outdoor environments and factors affecting their concentrations. Environ. Sci. Technol..

[55] Mancilla Y., Mendoza A., Fraser M.P., Herckes P. (2016). Organic composition and source apportionment of fine aerosol at Monterrey, Mexico, based on organic markers. Atmos. Chem. Phys..

[56] Offenberg J.H., Baker J.E. (1999). Aerosol size distributions of polycyclic aromatic hydrocarbons in urban and over water atmospheres. Environ. Sci. Technol..

[57] Sanderson A., Stott K., Stevens T.J., Thomas J.O. (2005). Engineering the structural stability and functional properties of the GI domain into the intrinsically unfolded GII domain of the yeast linker histone Hho1p. J. Mol. Biol..

