# Supplementary material for: Molecular Composition, Seasonal Variation, and Size Distribution of n-Alkanes, PAHs, and Saccharides in a Medium-Sized City of Guanzhong Plain, Northwest China: Evaluation of Control Measures Executed in the Past Decade
Source: Toxics. 2023 Feb 9;11(2):164. doi: 10.3390/toxics11020164 (PMC9959670; doi:10.3390/toxics11020164)
Supplement: Supplementary file 1 [file toxics-11-00164-s001.zip › toxics-2155383-supplementary.pdf]

Molecular Composition, Seasonal Variation, and Size Distribution of n-Alkanes, PAHs, and Saccharides in a Medium-Sized City of Guanzhong Plain, Northwest China: Evaluation of Control Measures Executed in the Past Decade

Bianhong Zhou [1,2,*], Qiao Feng [1,2], Chunyan Li [1], Lihua Jiao [1], Kaijing Cheng [1], Steven Sai Hang Ho [3], Zhongtao Wen [4] and Jianjun Li [2,*]

[1] College of Geography and Environment, Baoji University of Arts and Sciences, Shaanxi Key Laboratory of Disaster Monitoring and Mechanism Simulation, Baoji 721013, China
[2] State Key Laboratory of Loess and Quaternary Geology, Key Lab of Aerosol Chemistry and Physics, Institute of Earth Environment, Chinese Academy of Sciences, Xi'an 710061, China
[3] Division of Atmospheric Sciences, Desert Research Institute, Reno, NV 89512, USA
[4] Baoji Ecological Environment Science and Technology Service Center, Baoji 721000, China
* Correspondence: bhz620@163.com (B.Z.); lijj@ieecas.cn (J.L.)

Test S1

Student's *t*-test: Tests whether there is a significant difference in the means between the two sets of data. The calculation formula is as follows:

$$t = \frac{X_1 - X_2}{\sqrt{\frac{S_2^2}{n_1} + \frac{S_2^2}{n_1}}}$$

X: average value, *S*: standard deviation, *n*: the number of samples.

In this study, the Student's *t*-test was analyzed by SPSS software. The normality analysis showed that the P value of n-alkanes was greater than 0.05 during the day (p = 0.18) and at night (p = 0.11). The P value of PAHs was 0.18 during the day and 0.11 at night. It was indicated that the data have normality. The *t*-test showed that the *p* value of the day and night data was less than 0.01. In addition, using Excel (=TTEST) for further verification, the *p* values of n-alkanes and PAHs diurnal data were calculated to be 0.000031 and 0.000033, respectively, both less than 0.01. The results showed that there was a significant difference between the two sets of data at night and day.

Text SII

To describe the size distribution characteristics of the organic compounds, their GMD values were calculated using the following equation ([57]).

$$\log GMD = \sum C_i \log Dp_i \,/ \sum C_i$$

where C_i is the concentration of the target compounds collected at impactor stage *i* and Dp_i is the GMD of particles collected at impactor stage *i*.

Table S1. Meteorological parameters in Baoji city during the sampling periods.

Seasons		Temperature (℃)	Relative Humidity (%)	Atmospheric Pressure (hPa)	Wind Speed (m/s)
Spring (18 April 2017–8 May 2017)	Daytime	21.3	41.1	946	1.6
	Nighttime	17.0	58.4	946	1.4
	Average	19.2	49.7	946	1.5
Summer (15 July 2017–4 August 2017)	Daytime	32.9	46.6	937	1.5
	Nighttime	29.6	56.7	937	1.3
	Average	31.2	51.7	937	1.4
Autumn (15 October 2017–5 November 2017)	Daytime	15.2	70.4	955	1.2
	Nighttime	12.9	82.6	955	1.1
	Average	14.1	76.5	955	1.1
Winter (15 December 2017–2 January 2018)	Daytime	6.8	39.3	959	1.2
	Nighttime	2.8	51.0	960	1.4
	Average	4.9	45.1	960	1.3

Table S2. Diurnal concentrations of n-alkanes, PAHs, and saccharides in the four seasons (ng·m^{-3}) (the confidence interval represents the standard deviation).

	**Spring**		**Summer**		**Autumn**		**Winter**	
	**Day**	**Night**	**Day**	**Night**	**Day**	**Night**	**Day**	**Night**
**n-alkanes**								
octadecane (C18)	9.4 ± 0.8	10.6 ± 1.2	3.2 ± 0.33	8.6 ± 0.9	5.7 ± 0.43	8.7 ± 0.88	11.8 ± 0.15	13.0 ± 0.44
nonadecane (C19)	8.8 ± 0.64	9.0 ± 0.75	2.5 ± 0.15	8.6 ± 0.4	11.5 ± 0.59	13.5 ± 1.0	18.8 ± 0.20	17.8 ± 0.52
eicosane (C20)	7.0 ± 0.39	6.8 ± 0.32	3.2 ± 0.18	5.8 ± 0.2	7.6 ± 0.20	10.4 ± 0.39	21.0 ± 0.25	21.8 ± 0.50
henicosane (C21)	14.3 ± 0.68	14.0 ± 0.74	7.1 ± 0.46	12.6 ± 0.71	13.1 ± 0.61	17.9 ± 1.0	27.0 ± 1.5	32.7 ± 2.3
docosane (C22)	18.0 ± 0.94	17.9 ± 1.3	7.9 ± 0.79	10.3 ± 0.3	17.8 ± 0.71	19.9 ± 1.3	32.7 ± 3.1	44.7 ± 4.5
tricosane (C23)	21.6 ± 1.1	21.4 ± 1.6	13.9 ± 1.1	23.0 ± 1.4	21.7 ± 1.2	26.2 ± 2.1	38.1 ± 4.0	55.5 ± 6.1
tetracosane (C24)	23.3 ± 1.3	23.7 ± 2.1	18.7 ± 1.5	31.0 ± 2.4	26.3 ± 1.7	30.0 ± 2.7	32.6 ± 3.5	53.0 ± 6.0
pentacosane (C25)	27.1 ± 1.6	28.0 ± 2.4	26.2 ± 2.2	31.8 ± 2.2	30.5 ± 1.9	33.8 ± 3.1	35.4 ± 3.6	53.3 ± 5.8
hexacosane (C26)	20.9 ± 1.3	23.4 ± 2.3	20.7 ± 1.7	37.1 ± 3.9	26.5 ± 2.0	24.3 ± 2.2	21.8 ± 2.1	38.6 ± 4.1
heptacosane (C27)	28.7 ± 1.8	32.6 ± 2.6	24.2 ± 1.9	46.9 ± 4.7	36.5 ± 3.22	33.7 ± 2.9	28.4 ± 2.5	38.3 ± 4.1
octacosane (C28)	18.9 ± 1.1	21.2 ± 1.9	17.7 ± 1.2	36.9 ± 4.1	24.2 ± 1.8	21.5 ± 1.6	16.9 ± 1.17	27.2 ± 2.7
nonacosane (C29)	24.3 ± 1.2	29.4 ± 1.9	27.7 ± 1.3	44.4 ± 3.8	36.4 ± 2.1	41.3 ± 3.4	43.7 ± 3.4	48.2 ± 5.7
triacontane (C30)	25.9 ± 1.30	24.9 ± 1.3	23.5 ± 1.2	39.8 ± 2.8	27.8 ± 1.34	31.2 ± 1.7	18.4 ± 0.76	32.01 ± 3.1
hentriacontane (C31)	33.0 ± 1.7	35.2 ± 1.8	32.7 ± 1.5	46.8 ± 2.0	39.4 ± 1.78	43.2 ± 2.8	38.9 ± 2.3	41.3 ± 3.6
dotriacontane (C32)	30.0 ± 1.0	30.8 ± 1.9	27.7 ± 1.5	51.3 ± 3.5	32.8 ± 1.5	36.9 ± 1.7	18.9 ± 0.62	29.7 ± 2.3
tritriacontane (C33)	24.1 ± 0.87	29.6 ± 1.6	28.8 ± 1.7	54.6 ± 3.2	33.5 ± 1.8	36.55 ± 1.8	23.3 ± 0.99	26.6 ± 1.7
tetratriacontane (C34)	26.7 ± 2.4	34.7 ± 3.9	23.4 ± 1.6	44.3 ± 2.8	24.8 ± 1.1	27.2 ± 1.3	13.8 ± 0.45	19.4 ± 1.1
pentatriacontane (C35)	22.6 ± 2.8	26.5 ± 3.6	20.8 ± 1.5	41.9 ± 3.0	23.1 ± 1.9	27.3 ± 2.1	11.2 ± 0.42	14.6 ± 0.6
hexatriacontane (C36)	8.9 ± 0.44	11.9 ± 0.62	14.5 ± 1.3	35.6 ± 2.4	13.7 ± 0.80	14.7 ± 0.79	8.1 ± 0.36	12.4 ± 0.73
**Total n-alkanes**	391 ± 19.8	432 ± 31.8	344 ± 20.5	612 ± 42.4	453 ± 24.4	498. ± 32.1	462 ± 29.1	621 ± 50.7
**CPI**	1.2 ± 0.09	1.2 ± 0.12	1.2 ± 0.05	1.1 ± 0.08	1.2 ± 0.11	1.1 ± 0.10	1.3 ± 0.17	1.1 ± 0.11
**LMW**	129 ± 37.3	131 ± 62.6	82.7 ± 17.1	132 ± 105	134 ± 32.4	160 ± 26.3	218 ± 35.4	292 ± 75.4
**HMW**	261 ± 126	301 ± 62.7	262 ± 72.2	222 ± 14.5	319 ± 34.3	338 ± 59.5	244 ± 97.4	328 ± 82.6
**Plant wax**	44.9 ± 3.6	54.2 ± 4.6	35.4 ± 1.9	74.7 ± 5.6	61.9 ± 4.8	69.9 ± 5.9	83.2 ± 7.1	118 ± 15.5
**Fossil fuel**	346 ± 16.9	377.8 ± 27.7	309 ± 18.8	537.3 ± 37.3	391 ± 19.8	428.9 ± 26.6	378.3 ± 22.9	503 ± 36.9
**Saccharides**								
galactosan (Gala)	1.0 ± 0.13	3.54 ± 0.58	1.5 ± 0.12	1.9 ± 0.14	8.8 ± 0.98	14.4 ± 1.9	59.7 ± 6.4	80.4 ± 11.6
mannosan (Manno)	0.85 ± 0.11	3.05 ± 0.52	1.4 ± 0.15	1.9 ± 0.17	5.8 ± 0.69	10.8 ± 1.5	44.2 ± 4.9	54.5 ± 7.6
levoglucosan (Levo)	10.0 ± 1.8	34.33 ± 6.73	18.2 ± 2.5	22.6 ± 2.9	72.9 ± 9.2	133 ± 18.2	422 ± 46.6	529.7 ± 74.2
arabitol (Arab)	1.6 ± 0.14	4.39 ± 0.95	5.0 ± 0.30	6.5 ± 0.56	4.5 ± 0.29	4.8 ± 0.40	9.07 ± 0.45	9.9 ± 1.1
fructose (Fluc)	16.8 ± 1.	33.51 ± 5.11	6.1 ± 0.71	6.1 ± 0.54	4.72 ± 0.39	4.7 ± 0.48	12.3 ± 0.37	11.9 ± 1.0
glucose (Gluc)	26.1 ± 3.9	52.03 ± 7.62	18.9 ± 1.3	21.4 ± 1.5	9.9 ± 0.52	10.5 ± 0.59	15.3 ± 0.64	10.9 ± 0.64
mannitol (Manni)	1.5 ± 0.15	6.00 ± 1.15	8.5 ± 0.50	10.8 ± 0.80	4.0 ± 0.28	3.8 ± 0.26	4.0 ± 0.19	2.6 ± 0.16
inositol (Inos)	1.3 ± 0.22	1.44 ± 0.24	1.5 ± 0.26	1.4 ± 0.14	0.65 ± 0.06	0.76 ± 0.09	1.7 ± 0.17	1.6 ± 0.19
sucrose (Sucr)	9.9 ± 1.7	29.95 ± 6.05	8.4 ± 1.3	7.9 ± 1.1	2.3 ± 0.17	2.0 ± 0.13	14.7 ± 1.19	10.0 ± 0.76
trehalose (Treh)	0.9 ± 0.05	1.87 ± 0.27	3.9 ± 0.22	4.4 ± 0.31	1.3 ± 0.06	1.2 ± 0.09	3.5 ± 0.30	2.3 ± 0.22
**Total saccharides**	70.0 ± 8.0	170.13 ± 26.51	73.6 ± 5.6	84.9 ± 6.3	114 ± 11.8	186 ± 22.7	587 ± 56.8	714 ± 95.6
**PAHs**								
phenanthrene (Phe)	-[a]	-	-	-	-	-	6.5 ± 0.55	7.3 ± 0.87
anthracene (Ant)	-	-	-	-	-	-	0.25 ± 0.02	0.3 ± 0.02
fluoranthene (Flu)	-	-	-	-	-	-	7.5 ± 0.63	7.8 ± 0.65
pyrene (Pyr)	-	-	-	-	-	-	5.5 ± 0.57	6.3 ± 0.62

benz(a)anthracene (BaA)	-	-	-	-	-	-	3.5 ± 0.42	4.9 ± 0.62
chrysene/triphenylene (CT)	-	-	-	-	-	-	5.4 ± 0.62	6.1 ± 0.73
benzo(b/k)fluoranthene (BbkF)	-	-	-	-	-	-	10.1 ± 1.22	11.3 ± 1.4
benzo(e)pyrene (BeP)	-	-	-	-	-	-	4.1 ± 0.49	4.5 ± 0.56
benzo(a)pyrene (BaP)	-	-	-	-	-	-	3.1 ± 0.38	4.1 ± 0.52
perylene (Per)	-	-	-	-	-	-	0.62 ± 0.08	0.98 ± 0.12
indeno[123-cd]pyrene (IP)	-	-	-	-	-	-	4.3 ± 0.53	5.3 ± 0.67
dibenz(a,h)anthracene (DBA)	-	-	-	-	-	-	3.3 ± 0.41	4.2 ± 0.53
benzo(ghi)perylene (BghiP)	-	-	-	-	-	-	0.70 ± 0.09	0.89 ± 0.11
**Total PAHs**	-	-	-	-	-	-	54.8 ± 5.8	64.3 ± 7.1

[a] Concentrations in the impact samples of the three seasons are below the detection limit

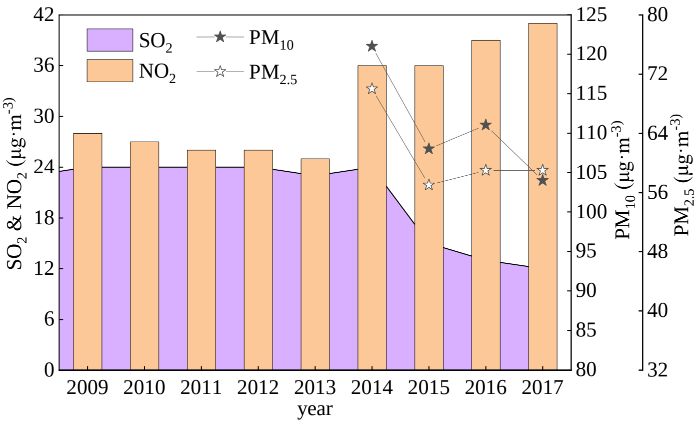

Figure S1. Interannual variation of SO_2, NO_2, and PM_{10} mass concentrations in Baoji city from 2008 to 2017. The data come from the 2008–2018 air quality bulletin of the Baoji Environmental Ecology Bureau (http://sthjj.baoji.gov.cn/art/2015/6/4/art_3733_287085.html).

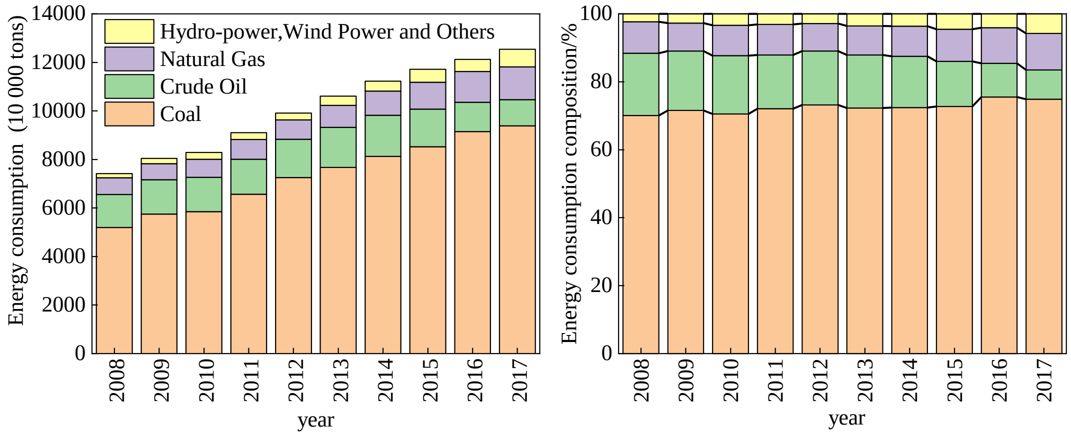

Figure S2. Interannual variation of energy consumption in Shaanxi Province

The data come from the Statistical Yearbook of Shaanxi Province from 2008 to 2018 (http://tjj.shaanxi.gov.cn/tjsj/ndsj/tjnj/).
